# Advances in Immunotherapy for Transplant Oncology

**DOI:** 10.3390/cancers16132369

**Published:** 2024-06-28

**Authors:** Maen Abdelrahim, Abdullah Esmail, Aiwu Ruth He, Moh’d Khushman, Yaser Rayyan

**Affiliations:** 1 Section of GI Oncology, Houston Methodist Neal Cancer Center, Houston, TX 77030, USA; aesmail@houstonmethodist.org; 2 Gastrointestinal Medical Oncology, Georgetown University Hospital, Washington, DC 20007, USA; 3 Division of Oncology, Washington University, St. Louis, MO 63110, USA; 4 Department of Gastroenterology & Hepatology, Faculty of Medicine, The University of Jordan, Amman 11942, Jordan

The increasing incidence of global cancer rates has created an entirely new demand for curative treatment modalities to improve patient outcomes. This is evident by the emergence of transplant oncology as a promising concept and a more prevalently utilized modality of liver cancer care. Aggressive malignancies of the liver, such as hepatocellular carcinoma (HCC) and cholangiocarcinoma (CCA), collectively account for the majority of all diagnosed primary liver malignancies [1,2,3,4]. An unfortunate mark of HCC and CCA is the asymptomatic development of the disease in its initial staging, leaving poor prognoses after advanced stages, limiting treatments, and revoking the curative resection viability [5,6]. Transplant oncology is still an emerging approach of treatment in the field of cancer care, but it has seen tremendous growth in improving the outcome of patients who underwent liver transplants for liver cancer, as indicated by the exponential spike in research over the past decade [7].

The utilization of immunotherapy (IO) has substantially shifted outcomes, as research clinicians apply and explore interventive therapies in various combinations and settings of treatment. The improved outcomes demonstrated in these studies using immune checkpoint inhibitors (ICPIs) [8,9] have encouraged transplant oncology researchers to investigate if ICPIs can be used to bridge more patients with HCC to liver transplant (LT) [10,11]. Recently, several reported cases have shown success in using ICPIs for downstaging tumors, resulting in the re-evaluation of patients with unresectable HCC for transplant after a successful downstage to LT criteria. Specific to hepatobiliary malignancies, the Milan Criteria stand as the universal golden rule following a study by Mazzaferro et al. in 1996 [12], indicating improved patient outcomes as a result of adherence to the strict LT guidelines. Since this important study, various international guideline criteria have been published that deliberately expand the parameters in a step-by-step manner to include a wider range of patients. Strict adherence to the LT criteria is not the sole barrier to increasing transplant capabilities, since the limited supply of organs is also a significant obstacle. In order to ameliorate the challenge of transplantable organ shortage, additional research on living donor liver transplantation (LDLT) in conjunction with deceased donor liver transplantation (DDLT) is warranted [13,14,15]. Additionally, clinicians have to consider survival and outcome improvements being specifically related to both the ICPI options available and the setting in which ICPIs are applied [16,17,18,19,20]. The updated guidelines from the ILTS—ILCA Consensus Conference in 2024 [21] will be reported in order to delineate the most contentious aspects of transplant oncology’s methodologies as well as to update/set safe parameters for downstaging criteria, macrovascular invasion, wash-out periods, recurrence care, ICPI setting utilization, and immunosuppressive interactions.

The pathology of HCC and CCA leaves most diagnosed patients undergoing treatment outside the designated hepatobiliary criteria set for LT [22,23,24]. Regardless of clinical opinions about treatment, the major focus during the ILCA Consensus Conference was patient candidacy for neoadjuvant ICPI utilization, which was determined to be viable for option individuals beyond LT criteria, with a high probability of meeting the Milan Criteria. For patients without extra-hepatic disease who do not achieve sufficient response to LRT, ICPIs might be used for downstaging, but additional clinical trials are needed. The conference discussions did not recommend a short waiting period after response to ICPI therapy to ensure no recurrence in patients who have been downstaged to center transplant criteria. Additionally, throughout the meeting, there was no clear recommendation given regarding ICPIs since the impact of ICPI used prior to liver transplant may impact graft rejection. After the completion of a specific ICPI regimen, the recommended washout period has been approximately 2–3 half-lives, or about 3 months, before LT. This designated “washout” period is an increasingly common and beneficial phase in transplant oncology that multidisciplinary trial teams are finding leads to better overall transplant outcomes [25]. Post-LT utilization of immunosuppressant (IS) has performed well in some published trials, but there is no direct evidence to corroborate that IS has direct causality in preventing or avoiding transplant rejection for patients who have previously received ICPI.

Patients who have confirmed HCC with macrovascular invasion (MVI) can be considered for LDLT after successful downstaging (no extrahepatic disease, an AFP cutoff, PET non-avid tumor) and response to downstaging therapy to ensure acceptable tumor biology. The ILCA Consensus Conference paid close attention to aspects of MVI in relation to LT treatments and the associated risks and benefits involved. The ILCA Consensus Conference emphasized that there is no strong evidence that supports the routine utilization of ICPI in the neoadjuvant setting for LT patients with confirmed MVI.

The post-LT setting of transplant oncology is the most significant step to ensure improved survival outcomes for participating patients. Recurrence surveillance and follow-up procedures are recommended at a minimum of every six months and should include cross-sectional contrast-enhanced imaging of the affected chest, abdomen, and pelvis. Additionally, the evaluation of serum alpha-fetoprotein (AFP) is recommended in high-risk individuals (based on RETREAT score [26]) with elevated pre-treatment serum AFP levels but there is no standardized timing of the intervals. Cases of recurrence have an unfortunate correlation to hepatobiliary malignancy treatment and recovery, which is directly related to the intense imaging follow-ups recommended under new ILCA guidelines. Post-LT recurrence recommendations are heavily reliant on clinicians strictly adhering to follow-up procedures so that early detection measures such as isolated resection, locoregional therapies, or radiotherapy can be implemented.

Standardization of structural concepts within the evolving field of transplant oncology is essential to the optimization of cancer care and patient management. The recent push for additional studies and the collection of comparative data highlights the current consensus. Furthermore, it is emphasized that multidisciplinary trial teams would significantly expedite the establishment of recommended criteria for the expansion of transplant eligibility. Studies performed by collaborative teams and the use of novel interventions have played a significant role in the recently improved survival outcomes in patients with hepatobiliary malignancies. Additional options now available in ICPI treatment create a novel opportunity to establish distinct options for patients and improve outcomes.
